# DC/TMD Axis II Instruments and Their Association with MRI-Based Structural Classification of the Temporomandibular Joint and Oral Function in Female Patients with Malocclusion: A Cross-Sectional Study

**DOI:** 10.3390/dj14100660

**Published:** 2026-10-10

**Authors:** Mao Katayama, Mari Kaneda, Yudai Shimpo, Rintaro Kubo, Akira Mishima, Chinami Igarashi, Hiroshi Tomonari

**Affiliations:** 1 Department of Orthodontics, Tsurumi University School of Dental Medicine, Yokohama 230-8501, Japan; katayama-m@tsurumi-u.ac.jp (M.K.); shinpo-y@tsurumi-u.ac.jp (Y.S.); kubo-r@tsurumi-u.ac.jp (R.K.); tomonari-h@tsurumi-u.ac.jp (H.T.); 2 Department of Diagnostic Imaging, Tsurumi University Dental Hospital, Yokohama 230-8501, Japan; mishima-a@tsurumi-u.ac.jp; 3 Department of Oral and Maxillofacial Radiology and Diagnosis, Tsurumi University School of Dental Medicine, Yokohama 230-8501, Japan; igarashi-c@tsurumi-u.ac.jp

**Keywords:** temporomandibular disorders, magnetic resonance imaging, DC/TMD, Axis II, Oral Behaviors Checklist, Jaw Functional Limitation Scale, disc displacement, osteoarthritis, occlusal function, female patients

## Abstract

**Background/Objectives:** Few studies have examined the associations of the Oral Behaviors Checklist (OBC) and the Jaw Functional Limitation Scale (JFLS) of the Diagnostic Criteria for Temporomandibular Disorders (DC/TMD) Axis II with magnetic resonance imaging (MRI)-based structural characterization of the temporomandibular joint, and most have analyzed these instruments only at the scale level. We examined these associations in female patients with malocclusion in a sample that was selected without symptom-based screening and independently selected under the eligibility criteria of the present study. **Methods:** In this cross-sectional study, 240 female patients with malocclusion who had undergone TMJ MRI irrespective of symptoms were classified according to MRI findings into four groups: an osteoarthritis (OA) group characterized by degenerative osseous changes in the mandibular condyle, bilateral disc displacement (BDD), unilateral disc displacement (UDD), and Osseous Change/Disc Displacement negative (OC/DD (−)) groups (n = 39, 89, 53, and 59, respectively). Individual items, scale scores, and five oral function parameters were compared using the Kruskal–Wallis test followed by Dunn’s post hoc test, as appropriate. The OBC total score and the JFLS score were the primary outcomes, whereas item-level and oral function analyses were exploratory. The JFLS was analyzed as a continuous variable; for the OBC, a total score ≥ 25 defined a high oral behavior score, and odds ratios (ORs) were estimated using logistic regression. A prespecified ordered trend was evaluated using the Jonckheere–Terpstra test. **Results:** The OBC total score differed significantly among the four groups (*p* = 0.033), whereas the four-group comparison of the JFLS score did not reach statistical significance (*p* = 0.052). The prespecified ordered-trend test was significant for both the OBC total score and the JFLS score (*p* = 0.011 and *p* = 0.016, respectively); however, inspection of the group-specific estimates indicated that these trends were driven mainly by the OA group rather than by a graded increase across all four categories. Compared with the OC/DD (−) group, the OA group had higher odds of a high oral behavior score (OR = 2.72, 95% confidence interval [CI] 1.11–6.70), whereas the UDD and BDD groups did not differ from the OC/DD (−) group. In exploratory analyses, awake clenching (*p* = 0.011), chewing hard bread (*p* = 0.047), chewing soft food (*p* = 0.036), and occlusal contact area (*p* = 0.045) differed significantly; awake clenching was significantly higher in the OA group than in both the OC/DD (−) and UDD groups, whereas chewing hard bread, chewing soft food, and occlusal contact area differed significantly only between the OA and OC/DD (−) groups. **Conclusions:** The OBC total score was higher in patients with MRI-defined degenerative osseous changes in the mandibular condyle, whereas no statistically significant differences were detected between either disc displacement group and the OC/DD (−) group. Item-level differences were derived from exploratory analyses that were not adjusted for multiple testing and should be regarded as hypothesis-generating. These cross-sectional findings suggest an association between degenerative osseous change in the TMJ and Axis II measures, but do not establish a graded relationship across structural categories or a temporal or causal relationship.

## 1. Introduction

Temporomandibular disorders (TMDs) are a group of musculoskeletal and neuromuscular conditions involving the temporomandibular joint (TMJ), the masticatory muscles, and related anatomical structures. They present with joint pain, joint sounds, tenderness of the masticatory muscles, and restricted mandibular movement, and may impair oral function and quality of life [1]. TMDs are widely recognized as multifactorial disorders involving structural, functional, psychological, hormonal, and behavioral components [2,3], and no single factor accounts for their onset or progression.

Epidemiological studies report a markedly higher prevalence of TMDs in females and young adults, and hormonal differences, psychological stress, and parafunctional behaviors have been suggested as contributors to these demographic disparities [2,3]. Accordingly, the present study focused exclusively on female patients to reduce sex-related heterogeneity. Associations between skeletal malocclusion or occlusal features and TMDs have also been reported [4], although recent systematic reviews have concluded that a causal relationship between occlusion and TMDs remains unsubstantiated [5].

Structural changes in the TMJ encompass abnormalities of both soft and hard tissues, including articular disc displacement and degenerative osseous changes in the mandibular condyle [6]. These findings, however, should not necessarily be interpreted as sequential stages of disease progression. Disc displacement is also highly prevalent among asymptomatic individuals in the general population, and most such joints have been reported to remain stable over time [7,8,9,10,11]. Imaging findings should therefore not be equated with a pathological state. Magnetic resonance imaging (MRI) permits noninvasive assessment of the position and morphology of the articular disc and can also depict osseous changes in the mandibular condyle, making it useful for the objective characterization of these structural features [12].

To account for the multifactorial nature of TMDs, the Diagnostic Criteria for Temporomandibular Disorders (DC/TMD) was developed as a standardized system for clinical and research use [1]. The DC/TMD comprises two complementary axes: Axis I, which addresses physical diagnoses, and Axis II, which assesses psychosocial and behavioral factors. The Oral Behaviors Checklist (OBC) and the Jaw Functional Limitation Scale (JFLS), both included in Axis II, assess the frequency of oral parafunctional behaviors and self-reported jaw functional limitation, respectively, and the reliability and validity of both instruments have been established [13,14,15,16,17,18].

Bruxism, which represents a subset of the oral behaviors assessed by the OBC, has been defined and graded by international consensus [19,20], and its prevalence in adults has been reported systematically [21]. Ecological momentary assessment has recently been introduced to assess awake bruxism and to reduce the recall bias inherent in questionnaires [22]. In prospective cohort studies, oral behaviors have emerged as predictors of first-onset TMD, with individuals scoring high on the OBC showing a substantially increased risk [23], and cross-sectional studies have likewise reported associations between individual oral behaviors and painful TMD [24,25]. Related work has examined sleep quality and causal mediation in TMD onset [26,27], and higher OBC scores have been reported in patients with greater pain-related disability [28].

Most existing studies relating the OBC and the JFLS to TMJ structure have, however, focused on scale-level scores. In such analyses, potentially informative item-specific associations may be obscured when responses are aggregated into scale scores. Chantaracherd et al. found no significant association between the JFLS total score and structural classification, yet reported that associations were suggested when the mastication and mobility subscales were examined separately [29]. This indicates that item-level analysis may reveal associations with structural involvement that are obscured at the scale level.

Our previous study demonstrated that MRI-defined structural categories—classified into four groups: OC/DD (−), UDD, BDD, and OA—were associated with oral function and craniofacial morphology, and identified the absence of a DC/TMD Axis II assessment as a limitation [30]. Furthermore, to our knowledge, no study has compared the OBC and the JFLS item by item in a group characterized by MRI-defined degenerative osseous changes in the mandibular condyle. The MRI-based classification used here represents structural characterization and should not be considered equivalent to diagnostic categorization based on DC/TMD Axis I.

Therefore, the primary purpose of this cross-sectional study was to examine whether the OBC total score and the JFLS score differ according to an MRI-based structural classification of the TMJ (OC/DD (−), UDD, BDD, and OA) in female patients with malocclusion. Secondary and exploratory objectives were to examine item-level differences in the OBC and the JFLS, their associations with oral function, and the proportion of patients with a high oral behavior score. The present study was conducted in the same department as our previous MRI-based investigation [30], but in a sample independently selected under the eligibility criteria of the present study, without symptom-based screening, with the novel objective of evaluating DC/TMD Axis II measures at both the item and scale levels. We hypothesized that the Axis II measures would be higher, and that some oral function parameters would be lower, in categories with greater structural involvement.

## 2. Materials and Methods

### 2.1. Study Design, Subjects, and Eligibility Criteria

**Figure 1:**
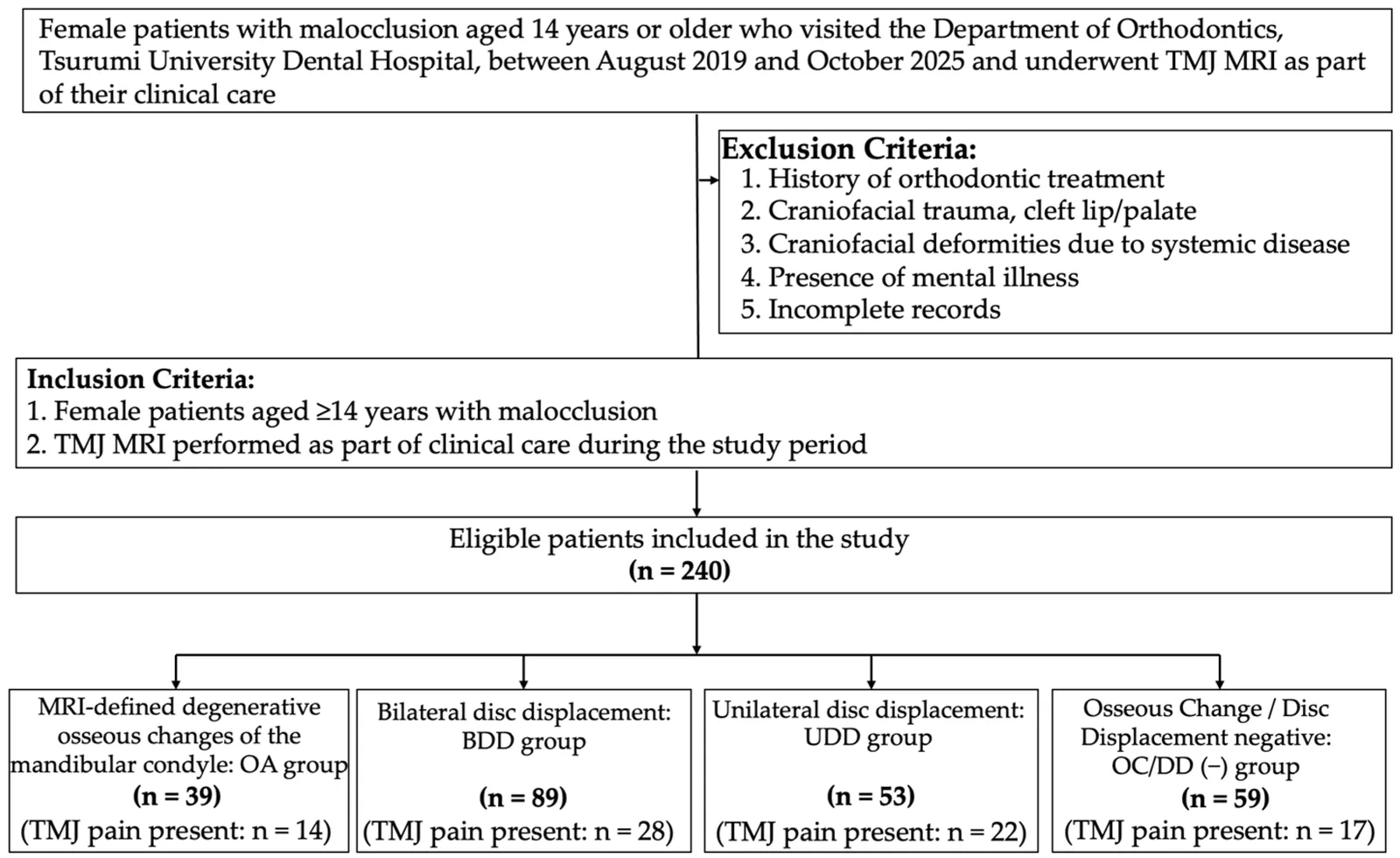
**Flow diagram of participant eligibility and MRI-based structural classification.** Eligible female patients with malocclusion were classified into four groups according to magnetic resonance imaging (MRI) findings: the osteoarthritis (OA) group, characterized by MRI-defined degenerative osseous changes in the mandibular condyle; the bilateral disc displacement (BDD) group; the unilateral disc displacement (UDD) group; and the Osseous Change/Disc Displacement negative (OC/DD (−)) group. The numbers of patients with temporomandibular joint (TMJ) pain in each group are also shown.

This observational study was conducted in accordance with the STROBE (Strengthening the Reporting of Observational Studies in Epidemiology) guidelines [31]. Among female patients aged 14 years or older who visited the Department of Orthodontics, Tsurumi University Dental Hospital, between August 2019 and October 2025, 240 female patients with malocclusion (mean age 26.1 ± 10.8 years) who met the inclusion criteria and underwent MRI examination of the temporomandibular joint as part of their clinical care were included. MRI was performed when clinically indicated, including as part of the preoperative assessment of patients scheduled for orthognathic surgery, in whom imaging was obtained irrespective of the presence of temporomandibular symptoms; these images were used retrospectively in the present analysis. The selection of subjects differed from that of our previous study [30]. In the previous study, the sample consisted of patients with clinical signs or symptoms suggestive of TMD, identified by a preliminary screening based on selected items of the DC/TMD Axis I protocol. In the present study, no symptom-based screening was applied: all female patients with malocclusion who visited the department during the study period and had undergone TMJ MRI for any clinical indication were eligible, including patients with dentofacial deformity scheduled for orthognathic surgery, in whom MRI was obtained as part of routine preoperative assessment irrespective of symptoms. The present sample therefore comprises both symptomatic and asymptomatic patients and a wider range of malocclusion, including dentofacial deformity, than the previous sample. It was selected independently under these criteria and does not constitute an extension of the previously reported cohort. Because the study periods overlap, some patients may have been included in both studies; however, individual patients were not cross-referenced with the previous cohort, and cohort membership was therefore not available as a variable in the present dataset (see Section 4). Exclusion criteria were a history of orthodontic treatment, craniofacial trauma or cleft lip/palate, craniofacial deformity due to systemic disease, the presence of mental illness, and incomplete records. The participant eligibility and MRI-based structural classification process is summarized in Figure 1. The study was approved by the Ethics Committee of Tsurumi University School of Dental Medicine, and written informed consent was obtained from all participants.

### 2.2. MRI Examination and Group Classification

MRI was performed using a 0.4 T open MRI system (APERTO Inspire, Fujifilm Medical, Tokyo, Japan) with a 10.0 cm TMJ surface coil. The position of the articular disc and the morphology of the mandibular condyle were interpreted in accordance with established MRI-based structural criteria [9,12], and patients were classified into four groups using the same framework as that used in our previous study [30]: the OC/DD (−) group (Osseous Change/Disc Displacement negative, *n* = 59), the UDD group (unilateral disc displacement, *n* = 53), the BDD group (bilateral disc displacement, *n* = 89), and the OA group (characterized by MRI-defined degenerative osseous changes in the mandibular condyle, *n* = 39). Osseous change was defined as the presence of flattening, erosion, osteophyte formation, or sclerosis of the mandibular condyle according to the Research Diagnostic Criteria for Temporomandibular Disorders (RDC/TMD) image analysis criteria [12] (Table 1). The term osteoarthritis is used throughout to denote this MRI-defined degenerative osseous change and does not represent a clinically or histologically confirmed diagnosis. TMJ pain was assessed using the examination procedures of the DC/TMD Axis I protocol, in the same manner as in our previous study [30], at the initial visit and before MRI interpretation. Pain on mandibular opening, pain on lateral and protrusive movements, and pain on palpation of the temporomandibular joint were examined. Only pain that the patient recognized as familiar was recorded as positive, and a patient was classified as having TMJ pain if familiar pain was elicited in any of these items. Pain intensity, laterality, and chronicity were not evaluated. Images were acquired by a radiological technologist at the Department of Diagnostic Imaging, Tsurumi University Dental Hospital, and were interpreted by the same board-certified oral radiologist as in our previous study [30], following an identical procedure. The structural classification formed part of the routine imaging report and was therefore performed independently of, and prior to, the Axis II and oral function analyses reported here. Because the classification was performed by a single experienced radiologist, intra- and inter-examiner agreement was not quantified; the diagnostic reliability of this 0.4 T protocol for identifying disc displacement and osseous change in the TMJ has been reported previously [32].

### 2.3. DC/TMD Axis II Assessment

The Japanese versions of the OBC (21 items, each scored 0–4) and the JFLS (20 items, each scored 0–10) were used for the DC/TMD Axis II assessment [33]. Both instruments were administered to all patients as part of the routine initial examination, that is, before the MRI images were interpreted and the structural classification was made. The OBC total score (range 0–84) was obtained by summing the 21 items. In accordance with the standard scoring procedure for the JFLS-20 [15,16], the JFLS score (range 0–10) was calculated as the mean of the 20 items.

### 2.4. Evaluation of Oral Function

Five oral function parameters were assessed, and the evaluation methods were identical to those described in our previous study [30]. Occlusal contact area and maximum occlusal force were measured using Dental Prescale II (GC Corporation, Tokyo, Japan) [34]. Masticatory performance was assessed using color-changeable chewing gum (Chew Check Gum, Oral Care Inc., Tokyo, Japan) [35,36]; after 60 chewing cycles, the color change (ΔE) was measured with a colorimeter (CR-20, Konica Minolta Inc., Tokyo, Japan), and the mean value obtained during free chewing was used for analysis. Tongue pressure was measured using the JMS tongue-pressure measuring device (JMS Co., Ltd., Hiroshima, Japan), and lip-closing force was measured using the Ripple-Kun device (Shofu Inc., Tokyo, Japan) [37]. Each parameter was measured three times by trained examiners, and the mean of the three measurements was used for analysis.

### 2.5. Statistical Analysis

The OBC total score and the JFLS score were defined a priori as the primary outcomes, whereas the item-level analyses, oral function analyses, and correlation analyses were considered exploratory. The dichotomized OBC analysis and the ordered-trend analyses were considered secondary analyses. The analysis was organized hierarchically. The primary analysis compared the OBC total score and the JFLS score among the four groups. The secondary analyses comprised the ordered-trend analyses based on the prespecified ordering of the MRI-based categories and the calculation of odds ratios for the OBC total score dichotomized according to a previously established criterion. The exploratory analyses comprised the four-group comparisons of individual OBC and JFLS items and of the oral function parameters, and the correlation analyses between the Axis II measures and the oral function parameters.

For the scale-level comparisons, the distribution of the OBC total score and the JFLS score in each group was first examined using skewness, the Shapiro–Wilk test, and the proportion of subjects scoring zero. Because normality could not be assumed or the distributions were markedly right-skewed, the Kruskal–Wallis test (*df* = 3) was used, followed where significant by Dunn’s post hoc test with Bonferroni correction; ε^2^ was reported as the effect size. In these analyses, which require a summed or averaged score, unanswered items (16 cells for the OBC and 11 cells for the JFLS, corresponding to 0.27% of 9840 cells) were treated as zero, and all 240 subjects were analyzed. This treatment may slightly underestimate the true scores, but the number of missing cells was small and was not unevenly distributed among the groups.

The JFLS is a continuous measure of the degree of jaw functional limitation, and no clinically established cut-off point exists; it was therefore analyzed without dichotomization, using the score itself. For the OBC, the total score was compared among the four groups as a continuous variable in the main analysis. In addition, as a secondary analysis intended to interpret the magnitude of the between-group difference against a previously reported risk criterion, a total score of 25 or more was defined as a high oral behavior score in accordance with the DC/TMD scoring manual and the OPPERA studies [23,38]; odds ratios (ORs) and 95% confidence intervals (CIs) were then estimated by binary logistic regression with this dichotomous variable as the dependent variable and group as a set of dummy variables, with the OC/DD (−) group as the reference. This dichotomized analysis is based on the same data as the main analysis of the total score; it is not an independent verification, and its purpose is to translate the difference obtained in the main analysis into a clinically interpretable metric.

For the ordered analysis, the four MRI-based structural categories were arranged a priori as OC/DD (−), UDD, BDD, and OA according to increasing structural involvement; this ordering was used for statistical testing and should not be interpreted as a longitudinal sequence of disease progression. To test the prespecified hypothesis that the Axis II measures increase across these ordered categories, the Jonckheere–Terpstra test for ordered alternatives [39,40] was performed as a secondary analysis. A trend test was likewise applied to the proportion of subjects with a high oral behavior score by entering group as an ordinal score (1–4) in a logistic regression model. These analyses based on an ordered hypothesis were specified before the analysis began and were not selected post hoc. The scale-level analyses were performed using Python version 3.14.7 (SciPy version 1.18.1, statsmodels version 0.15.0, and scikit-posthocs version 0.16.1), and the significance level was set at 5%.

For the item-level comparisons, the Kruskal–Wallis test (*df* = 3) was used, and for variables that reached significance, pairwise comparisons were performed using Dunn’s post hoc test with Bonferroni correction for six comparisons. Epsilon squared (ε^2^) was calculated as the effect size. Because a large number of item-level comparisons were performed, these analyses were treated as exploratory, and no multiplicity adjustment was applied across the full set of OBC and JFLS items; therefore, item-level findings were interpreted cautiously. The association between the Axis II measures and the oral function parameters was examined using Pearson’s product-moment correlation coefficient (r). The correlation analyses were likewise exploratory and were not adjusted for multiple testing. The distribution of TMJ pain among the groups was compared using Pearson’s chi-squared test. In the item-level analysis, unanswered items were excluded only from the analysis of that item (236–240 subjects per item), and no imputation was performed. The significance level was set at *p* < 0.05, and effect sizes were interpreted using benchmarks specific to temporomandibular joint and masticatory muscle research [41]. The item-level analyses were performed using SPSS Statistics version 27.0 (IBM, Tokyo, Japan). The dataset analyzed in this study is provided in File S1.

## 3. Results

### 3.1. Demographic Characteristics

The numbers of subjects in the four groups were 59 in the OC/DD (−) group, 53 in the UDD group, 89 in the BDD group, and 39 in the OA group. No significant difference in age was observed among the four groups (Kruskal–Wallis H = 1.686, df = 3, *p* = 0.640; Table 2).

### 3.2. TMJ Pain Prevalence

The proportion of subjects with TMJ pain was 28.8% in the OC/DD (−) group, 41.5% in the UDD group, 31.5% in the BDD group, and 35.9% in the OA group, and no significant difference was observed among the four groups (χ^2^ = 2.359, df = 3, *p* = 0.501; Table 2). Although TMJ pain prevalence did not differ significantly among the groups, pain intensity and chronicity were not evaluated.

### 3.3. Primary Analysis: Scale-Level Intergroup Comparison of the OBC Total Score and the JFLS Score

The OBC total score and the JFLS score were compared among the four groups as scale-level measures. Skewness of the OBC total score ranged from 0.03 to 0.78, indicating a relatively symmetrical distribution, but normality was rejected by the Shapiro–Wilk test in the OC/DD (−) group (*p* = 0.043) and the BDD group (*p* = 0.003). The JFLS score was markedly right-skewed in all groups (skewness 1.04–2.51), and normality was rejected in every group (all *p* < 0.001). The proportion of subjects scoring zero, that is, reporting no jaw functional limitation at all, was 45.8% in the OC/DD (−) group, 35.8% in the UDD group, 30.3% in the BDD group, and 23.1% in the OA group. Nonparametric methods were therefore used for both measures.

The median [interquartile range] of the OBC total score was 18 [14–22] in the OC/DD (−) group, 19 [14–24] in the UDD group, 19 [14–23] in the BDD group, and 22 [17.5–29.5] in the OA group, and a significant difference was found among the four groups (Kruskal–Wallis *H* = 8.719, *df* = 3, *p* = 0.033, ε^2^ = 0.024). The only pairwise comparison that reached significance with Dunn’s post hoc test and Bonferroni correction was that between the OA group and the OC/DD (−) group (adjusted *p* = 0.027); the comparisons between the OA group and the UDD group (adjusted *p* = 0.130) and between the OA group and the BDD group (adjusted *p* = 0.144) did not reach significance.

The median [interquartile range] of the JFLS score (0–10) was 0.10 [0–0.78] in the OC/DD (−) group, 0.40 [0–1.20] in the UDD group, 0.25 [0–1.05] in the BDD group, and 0.70 [0.08–1.85] in the OA group. The overall four-group comparison did not reach statistical significance (Kruskal–Wallis *H* = 7.710, *df* = 3, *p* = 0.052, ε^2^ = 0.020; Table 3).

### 3.4. Secondary Analyses: High Oral Behavior Score and Prespecified Ordered Trends

The proportion of subjects with an OBC total score of 25 or more was 20.3% (12/59) in the OC/DD (−) group, 17.0% (9/53) in the UDD group, 22.5% (20/89) in the BDD group, and 41.0% (16/39) in the OA group. In the logistic regression with the OC/DD (−) group as the reference, the odds ratio for the OA group was 2.72 (95% CI 1.11–6.70, *p* = 0.029). By contrast, the odds ratios for the UDD group (0.80, 95% CI 0.31–2.09, *p* = 0.650) and the BDD group (1.14, 95% CI 0.51–2.54, *p* = 0.758) were not significant, and their confidence intervals included unity (Table 4). When group was entered as an ordinal score, the odds of a high oral behavior score were 1.37 times higher for each one-category increase in structural involvement (95% CI 1.02–1.85, *p* = 0.037). However, the proportions of subjects with a high oral behavior score in the OC/DD (−), UDD, and BDD groups (20.3%, 17.0%, and 22.5%, respectively) were similar, and only the OA group (41.0%) differed from the reference group; the significant trend statistic therefore reflects mainly the contrast between the OA group and the other three groups rather than a monotonic increase across all four categories.

The Jonckheere–Terpstra test based on the prespecified ordered hypothesis showed a significant increasing trend across the prespecified ordered categories for both the OBC total score (*z* = 2.548, *p* = 0.011) and the JFLS score (*z* = 2.415, *p* = 0.016; Table 3).

### 3.5. Exploratory Analyses: Item-Level Intergroup Comparisons and Oral Function

The individual OBC and JFLS items and the oral function parameters were compared among the four groups. The following item-level comparisons were exploratory and were not adjusted for multiple testing across the full set of OBC and JFLS items. A significant difference was found for awake clenching (OBC item 4; Kruskal–Wallis H = 11.211, df = 3, *p* = 0.011, ε^2^ = 0.035). In the pairwise comparisons using Dunn’s post hoc test with Bonferroni correction, the OA group (1.21 ± 1.06) scored significantly higher than the OC/DD (−) group (0.61 ± 0.89; adjusted *p* = 0.017) and the UDD group (0.60 ± 0.88; adjusted *p* = 0.018), whereas the difference from the BDD group (0.80 ± 1.01) was not significant (adjusted *p* = 0.138).

For the JFLS, significant differences were found for chewing hard bread (item 2; H = 7.961, *p* = 0.047, ε^2^ = 0.021) and chewing soft food (item 5; H = 8.571, *p* = 0.036, ε^2^ = 0.024). In both cases, the OA group (item 2: 3.36 ± 3.54; item 5: 0.79 ± 1.91) scored significantly higher, indicating greater functional limitation, than the OC/DD (−) group (item 2: 1.58 ± 2.49; item 5: 0.27 ± 1.10; adjusted *p* = 0.042 and 0.036, respectively), whereas the differences from the UDD and BDD groups were not significant.

Among the oral function parameters, a significant difference was found for occlusal contact area (H = 8.064, *p* = 0.045, ε^2^ = 0.021); the OA group (11.59 ± 5.90 mm^2^) had a significantly smaller area than the OC/DD (−) group (17.08 ± 10.57 mm^2^; adjusted *p* = 0.027). Maximum occlusal force was lower in the OA group than in the OC/DD (−) group (468.95 ± 185.98 N vs. 631.36 ± 337.33 N) but did not differ significantly among the four groups (H = 5.623, *p* = 0.131, ε^2^ = 0.011; OA vs. OC/DD (−), adjusted *p* = 0.114). Occlusal contact area and maximum occlusal force were strongly correlated (Pearson r = 0.934, *p* < 0.001). Occlusal force per unit contact area (occlusal force divided by occlusal contact area) was 39.59 ± 8.72 N/mm^2^ in the OC/DD (−) group, 41.23 ± 7.72 N/mm^2^ in the UDD group, 40.64 ± 7.39 N/mm^2^ in the BDD group, and 42.80 ± 9.60 N/mm^2^ in the OA group, with no significant difference among the four groups (H = 3.895, *p* = 0.273). No significant differences among the groups were found for masticatory performance (*p* = 0.777), tongue pressure (*p* = 0.530), or lip-closing force (*p* = 0.677). No significant differences were found for jaw thrusting (OBC item 7; *p* = 0.095), unilateral chewing (OBC item 16; *p* = 0.104), or opening the mouth wide enough to drink (JFLS item 7; *p* = 0.068) (Table 5). The complete results for all OBC and JFLS items and all oral function parameters are provided in Supplementary Table S1.

### 3.6. Exploratory Correlations Between Axis II Measures and Oral Function

Table 6 lists the OBC and JFLS items that showed a statistically significant unadjusted correlation (*p* < 0.05) with at least one oral function parameter; this selection was made post hoc on the basis of statistical significance in the present data and not on the basis of prespecified clinical hypotheses. The complete correlation matrix for all 21 OBC items and 20 JFLS items is provided in Supplementary Table S2. The exploratory correlation analyses showed that JFLS item 3 (chewing chicken) was negatively correlated with occlusal contact area (r = −0.24, *p* < 0.01) and maximum occlusal force (r = −0.21, *p* < 0.01), and JFLS items 1, 2, and 5 likewise showed weak but significant negative correlations with both parameters (|r| = 0.14–0.17). Among the OBC items, item 15 (holding the jaw with the hand, such as leaning on the chin) was positively correlated with maximum occlusal force (r = 0.17, *p* < 0.01) and occlusal contact area (r = 0.14, *p* < 0.05) and negatively correlated with lip-closing force (r = −0.14, *p* < 0.05), and item 1 (grinding or clenching the teeth during sleep) was positively correlated with masticatory performance (ΔE; r = 0.18, *p* < 0.01). Weak correlations with lip-closing force were also observed for OBC items 13 and 19 and JFLS item 6 (|r| = 0.13–0.26). Tongue pressure showed no significant correlation with any item (Table 6).

## 4. Discussion

The principal finding of this study, based on the prespecified primary outcomes, was that the OBC total score differed among the MRI-based structural groups and was higher in the OA group, characterized by MRI-defined degenerative osseous changes in the mandibular condyle, than in the OC/DD (−) group, whereas the JFLS score did not differ significantly in the overall four-group comparison; no statistically significant differences from the OC/DD (−) group were detected in either the unilateral or the bilateral disc displacement group. The exploratory item-level analyses suggested that these differences were most evident for awake clenching, the JFLS mastication items, and occlusal contact area in the OA group; however, these item-level analyses involved a large number of comparisons without adjustment for multiple testing, and the resulting findings are presented as hypothesis-generating observations, with an increased risk of false-positive results, that require confirmation in independent samples. That neither disc displacement subgroup differed from the OC/DD (−) group is consistent with the interpretation that statistically detectable differences were largely limited to the group with degenerative osseous changes; the absence of detectable differences does not, however, establish that no differences exist. This pattern is consistent with our previous study, which likewise identified reduced oral function only in the OA group using the same four-group classification [30]. Although the prevalence of TMJ pain did not differ significantly among the groups (*p* = 0.501), residual confounding by pain cannot be excluded, because pain intensity and chronicity were not evaluated.

Awake clenching, which was significantly higher in the OA group, is a behavior that imposes sustained mechanical loading on the TMJ. The observed association is biologically plausible, because sustained mechanical loading may contribute to degenerative change. However, the reverse explanation—that structural alteration, or symptoms associated with it, modifies oral behavior—cannot be excluded, and the present cross-sectional design does not allow the direction of the association to be determined. Finite element analyses have shown that prolonged clenching generates strong compressive stress in the condylar cartilage [42,43] and that protrusive or lateral movement of the mandible and rotation of the condyle produce eccentric stress on the articular disc [44,45,46]. Such abnormal mechanical loading may promote degeneration through damage to chondrocytes and the extracellular matrix [47]. Imaging studies have likewise reported associations between chewing side preference or a deviated mandibular position and degenerative joint changes [48,49], and jaw thrusting is epidemiologically associated with painful TMD [24]. These findings accord with reviews proposing that sustained loading exceeding the adaptive capacity of the joint leads to degeneration [50]. The higher frequency of awake clenching observed in the OA group suggests that the association between oral behaviors and TMJ structure may differ according to structural involvement. Jaw thrusting (OBC item 7) and unilateral chewing (OBC item 16) showed the highest mean values in the OA group but did not reach significance in the four-group comparison.

For the JFLS, the mastication items (chewing hard bread and chewing soft food) were significantly higher in the OA group than in the OC/DD (−) group. Chantaracherd et al. found no significant association between the total score and structural classification, yet reported that associations were suggested in the mastication and mobility subscales [29]. Consistent with this observation, the present exploratory item-level analysis identified differences in two mastication items, suggesting that item-level analysis may provide information that is not apparent from the overall scale score. Furthermore, the JFLS mastication items showed negative correlations with occlusal contact area and maximum occlusal force but no correlation with masticatory performance (ΔE), suggesting that these items may more closely reflect occlusal contact and force than the dynamic capacity to break down food. Masticatory performance assessed with color-changeable chewing gum principally evaluates mixing ability and does not necessarily correlate with occlusal force [36,51], and pain has also been reported to influence the JFLS mastication items [52]; the JFLS therefore may reflect multiple functional and pain-related aspects rather than a single dimension of oral function. By contrast, the correlations with lip-closing force were weak and inconsistent in direction, and they should be interpreted with caution given the exploratory nature of the correlation analyses.

With respect to oral function, occlusal contact area was significantly smaller in the OA group, and maximum occlusal force was approximately 26% lower in the OA group than in the OC/DD (−) group, although the four-group comparison did not reach statistical significance. These two measures are related quantities derived from the same pressure-sensitive film and were strongly correlated in the present data (r = 0.934). It therefore cannot be concluded that occlusal contact area alone differed between the groups while occlusal force was preserved. Maximum occlusal force showed a similar between-group pattern, although the four-group comparison was not statistically significant and substantial interindividual variability was observed. Indeed, in our previous study [30], both maximum occlusal force and occlusal contact area were significantly lower in the OA group, and the present findings are consistent in direction with that result.

Occlusal force per unit contact area did not differ significantly among the groups (OC/DD (−) group 39.59 ± 8.72 N/mm^2^, OA group 42.80 ± 9.60 N/mm^2^; *p* = 0.273). This finding suggests that the ratio of occlusal force to contact area was broadly similar across the MRI-based structural categories, despite the smaller occlusal contact area observed in the OA group. Because occlusal force and occlusal contact area were derived from the same pressure-sensitive film and were highly correlated (r = 0.934), this force-per-area analysis should be regarded as exploratory and does not permit mechanistic interpretation of the relationship between occlusal contact area and occlusal force. It should also be noted that the effect sizes for the significant item-level and occlusal contact-area comparisons were small (ε^2^ = 0.021–0.035), and cautious interpretation is required.

By contrast, neither disc displacement group showed the pattern of significant differences from the OC/DD (−) group observed for the OA group. In our previous study using the same four-group classification [30], reduced oral function was likewise confined to the OA group. Kaneda et al. interpreted the absence of a significant reduction in oral function in the UDD group as being consistent with the report of Sato et al., who found no marked decrease in occlusal force or masticatory performance in cases of unilateral joint abnormalities [53]. The present findings likewise indicate that the behavioral and functional differences identified in the item-level OBC and JFLS measures and oral function were predominantly observed in comparisons involving the OA group rather than the disc displacement groups. These findings suggest that disc displacement alone may not necessarily be accompanied by detectable differences in these measures, whereas such differences were more evident in the OA group. The difference between unilateral and bilateral displacement was not apparent in the oral function or Axis II measures examined here, while it may be detectable in skeletal morphology. The fact that disc displacement is also highly prevalent among asymptomatic individuals in the general population and that most such joints remain stable over time [7,10,11] is likewise consistent with the present finding of relatively limited behavioral or functional differences associated with the disc displacement groups. Nevertheless, higher oral behavior scores than in healthy individuals have been reported in patients with disc displacement without reduction [54,55,56], and a different pattern of association might be found if disc displacement were subdivided into displacement with and without reduction. With respect to craniofacial morphology, our previous study [30] found that the BDD group, like the OA group, had significantly larger ANB and FMA angles, suggesting that bilateral joint involvement may show a distinct association with craniofacial morphology even when corresponding differences in the Axis II or oral function measures are not detected.

A broadly similar pattern was observed when the OBC and the JFLS were examined as scale-level measures. The OBC total score differed significantly among the four groups, with a significant pairwise difference between the OA and OC/DD (−) groups, whereas neither disc displacement group differed significantly from the OC/DD (−) group. For the JFLS, the overall four-group comparison did not reach statistical significance, although the prespecified ordered-trend test was significant, as discussed below. That the item-level and scale-level analyses pointed in a broadly similar direction supports the overall observation that statistically detectable differences in the Axis II measures were most evident in the group with degenerative osseous changes in the mandibular condyle, although the strength of statistical evidence differed between the OBC and the JFLS. This partial dissociation between MRI-based structural findings and self-reported functional limitation accords with the report of Chantaracherd et al., who found no significant association between structural classification and the JFLS total score [29], and supports the view that an abnormality on imaging does not in itself signify a functional problem. A recent retrospective study by Brandsma et al. [57] of 202 patients with TMD who underwent TMJ MRI likewise found that psychological indices were dissociated from imaging findings: higher anxiety and depression scores were associated with greater perceived pain, whereas abnormal MRI findings were predicted by clinical signs such as unilateral involvement, locking, and clicking, and higher anxiety scores were, if anything, associated with normal joint morphology. Although that study assessed psychological distress rather than oral behaviors and functional limitation, and included both sexes and a broader clinical population, its findings and ours converge on the view that Axis II measures and MRI-based structural findings capture different dimensions of TMD and that structural findings on imaging should not be assumed to reflect the psychosocial or behavioral status of the patient. In the present study, the only structural category in which Axis II measures differed was the group with degenerative osseous change, which suggests that the relationship between behavioral measures and imaging findings may depend on the type and extent of structural involvement.

The analysis of odds ratios based on a dichotomized OBC total score is derived from the same data as the main analysis of the total score and should not be regarded as an independent finding. Its value lies in translating the magnitude of the between-group difference obtained in the main analysis into a form that can be interpreted against a previously reported risk criterion. The classification of an OBC total score of 25 or more as a high oral behavior score derives from the DC/TMD scoring manual and the OPPERA studies [23,38]. In the OPPERA prospective cohort, individuals with high OBC scores were shown to have a significantly increased risk of first-onset TMD [23]. In the present study, the odds of a high oral behavior score were 2.72 times higher in the OA group than in the OC/DD (−) group (95% CI 1.11–6.70), and the trend odds ratio across the prespecified ordered categories was 1.37 (95% CI 1.02–1.85). The trend estimate should be interpreted with caution. A trend test evaluates a prespecified ordered alternative and can reach statistical significance when only the highest category differs from the others; in the present data, the proportions of subjects with a high oral behavior score were similar in the OC/DD (−), UDD, and BDD groups (17.0–22.5%), and the significant trend was driven mainly by the OA group (41.0%). These findings therefore indicate an association between degenerative osseous change and higher oral behavior scores, but they do not provide evidence of a graded or stepwise increase across the disc displacement categories, and they should not be interpreted as evidence that oral behaviors precede structural changes. The present study is cross-sectional, and the direction of the association cannot be determined; structural alterations or associated symptoms may themselves influence oral behaviors or their self-reported frequency. These possibilities are not mutually exclusive, and the finding should therefore be described as an association rather than as evidence of causation. In addition, the OA group comprised 39 subjects and the confidence interval for the OA-group odds ratio was wide (1.11–6.70); the magnitude of the point estimate should therefore be interpreted cautiously.

For the JFLS, the overall four-group comparison did not reach statistical significance (Kruskal–Wallis test, *p* = 0.052). In contrast, the prespecified Jonckheere–Terpstra test based on the ordered categories OC/DD (−) → UDD → BDD → OA showed a significant trend (*p* = 0.016). These results should be interpreted according to the different hypotheses tested: the Kruskal–Wallis test evaluates differences among the four groups without assuming an order, whereas the Jonckheere–Terpstra test evaluates the prespecified ordered alternative. In addition, the proportion of subjects reporting no jaw functional limitation (JFLS score of zero) was 45.8% in the OC/DD (−) group, 35.8% in the UDD group, 30.3% in the BDD group, and 23.1% in the OA group. Taken together, these findings suggest an association between MRI-defined degenerative osseous change and greater jaw functional limitation at the group level; however, the evidence from the overall four-group comparison was not statistically significant, and the significant trend statistic should not be taken as evidence of a progressive increase across the disc displacement categories. Because no established diagnostic cut-off exists for the JFLS, the score was appropriately analyzed as a continuous measure without dichotomization.

Several points should be considered when interpreting this ordered trend. First, the median JFLS score was 0.40 in the UDD group and 0.25 in the BDD group, indicating that the group medians did not follow a strictly monotonic pattern. The significant Jonckheere–Terpstra test reflects the rank distributions across all four groups and does not require the group medians themselves to increase monotonically; a significant result can arise when the highest category alone differs from the others, as appears to be the case here. This finding therefore indicates that the distinction between unilateral and bilateral disc displacement did not correspond to a gradation in the JFLS score, and that the ordered trend was attributable mainly to the OA group. Second, the JFLS showed a substantial floor effect, with 23–46% of subjects scoring zero, which may limit the ability of summary measures such as the median to distinguish among groups. The present findings therefore support an association between the MRI-based structural categories and JFLS scores at the group level, but the observed differences were small and should not be used to infer the structural condition of an individual patient.

From a clinical perspective, the present findings may be relevant to the assessment of oral behaviors in patients with malocclusion. These clinical considerations are based on exploratory item-level findings that were not adjusted for multiple testing and should be regarded as preliminary. Awake clenching, which was more frequent in the OA group, is a potentially modifiable oral behavior that can be addressed through behavioral approaches. Oral behaviors have been reported as predictors of first-onset TMD [2,23], and behavioral modification is included as an important component of conservative self-management for TMD [58,59]. Assessment of oral behaviors may therefore be useful for identifying patients with frequent potentially unfavorable loading behaviors. However, longitudinal studies are required to determine whether modification of these behaviors is associated with a lower risk of subsequent structural changes in the TMJ. The JFLS mastication items may also provide clinically useful information regarding self-reported limitations during mastication, particularly when instrumental assessment of oral function is not available. Conversely, the relatively limited differences observed in the disc displacement groups compared with the OA group suggest that disc displacement on imaging alone should not be assumed to indicate corresponding behavioral or functional impairment. This may be useful when explaining MRI findings to patients. It should be emphasized, however, that this is a cross-sectional study, that these findings do not demonstrate causality, and that they do not constitute grounds for diagnosis or treatment decisions.

Several limitations should be considered. First, as a cross-sectional study, causal relationships cannot be inferred. Second, TMJ pain was recorded as a dichotomous variable, without distinguishing arthralgia from myalgia, and pain intensity and chronicity were not evaluated; the analysis was not stratified by pain status, and because the JFLS is also influenced by pain, the potential influence of pain on the observed associations cannot be fully excluded [52]. Third, disc displacement was not subdivided into displacement with and without reduction, so subtype-specific associations may have gone undetected [54]. Fourth, although computed tomography provides superior visualization of osseous changes, a 0.4 T open MRI system was used for the structural assessment in the present study, and the sensitivity for detecting subtle osseous changes may therefore have been limited [12]; in addition, the structural classification was performed by a single radiologist, so intra- and inter-examiner agreement was not quantified. Fifth, the subjects were exclusively female patients with malocclusion attending an orthodontic clinic, and the results cannot necessarily be extrapolated to the general population [5]. In addition, the selection mechanism differed from that of our previous study [30]: whereas MRI was previously performed only after symptom-based screening, the present sample included all female patients with malocclusion who had undergone TMJ MRI without symptom-based screening, including asymptomatic patients and patients with dentofacial deformity. The present sample is therefore a different population from the previously reported cohort and should not be regarded as an extension of it. Because the study periods overlap, some patients may have been included in both studies, but individual patients were not cross-referenced with the previous cohort; cohort membership could therefore not be included as a covariate, and a sensitivity analysis examining whether the associations were consistent between the two selection mechanisms could not be performed; a direct comparison with the previous findings is therefore not possible, and the possibility that the symptom-independent selection influenced the magnitude of the observed associations (selection or spectrum bias) cannot be excluded. The spectrum of structural involvement in this sample should also be regarded as specific to an orthodontic clinical setting that includes patients with dentofacial deformity. Because all subjects had malocclusion, the present data cannot determine whether the association between oral behaviors and degenerative osseous change is modified by malocclusion status, that is, whether a similar association would be observed in individuals without malocclusion. Occlusal factors have been proposed to influence loading of the TMJ, although a causal relationship between occlusion and TMD has not been established [5]; the possibility that malocclusion acts as an effect modifier of the observed association therefore remains an open question. Sixth, only the OBC and the JFLS were evaluated among the Axis II instruments, and measures of pain intensity and psychological distress were not included. Seventh, the OA group comprised only 39 subjects, and subdividing the disc displacement group into unilateral and bilateral forms reduced the number of subjects in each group, which may have limited the statistical power of some intergroup comparisons. Eighth, the analyses were not adjusted for potential confounders such as age, and residual confounding by unmeasured or uncontrolled factors cannot be excluded. Ninth, the item-level, oral function, and correlation analyses were exploratory and were not adjusted for multiple testing across the full set of comparisons; given the number of comparisons performed, these findings carry an increased risk of false-positive (chance) associations, should be regarded as hypothesis-generating rather than confirmatory, and require confirmation in independent samples with prespecified item-level hypotheses. In the scale-level analyses, unanswered items were treated as zero, which may slightly underestimate the true scores; because missing items were excluded in the item-level analyses, the numbers of subjects analyzed do not correspond exactly between the two. Future work should include a comprehensive Axis II assessment incorporating pain and psychological distress measures, subtype classification of disc displacement, longitudinal designs, and more detailed imaging assessment, together with an integrated analysis in the same population of the Axis II measures reported here and the objective oral function and craniofacial morphology reported previously [30]. Studies including individuals without malocclusion are also needed to determine whether the association between oral behaviors and degenerative osseous change observed here is specific to patients with malocclusion.

## 5. Conclusions

In female patients with malocclusion, the OBC total score, a prespecified primary outcome, differed according to the MRI-based structural classification of the TMJ and was higher in patients with MRI-defined degenerative osseous changes in the mandibular condyle (OA group) than in patients without disc displacement or osseous change; the overall four-group comparison of the JFLS score, the other primary outcome, did not reach statistical significance. In a secondary analysis using a previously reported criterion (a total score of 25 or more), the odds of a high oral behavior score in the OA group were 2.72 times those in the OC/DD (−) group. The prespecified ordered-trend tests were significant for both measures, but these trends were driven mainly by the OA group and do not indicate a graded increase across the disc displacement categories; no statistically significant differences were detected in either the unilateral or the bilateral disc displacement group, although this absence of detectable differences does not establish that no differences exist. Exploratory item-level analyses suggested that awake clenching, mastication-related jaw functional limitation, and occlusal contact area differed in the OA group; because these analyses involved a large number of comparisons without adjustment for multiple testing, they should be regarded as hypothesis-generating findings with an increased risk of false-positive results, rather than as confirmatory evidence. The effect sizes were small, and the cross-sectional design precludes inference regarding temporal or causal relationships. Because temporomandibular disorders are of multifactorial etiology, these findings should be interpreted in conjunction with clinical findings and psychosocial assessment.

## Figures and Tables

**Table 1:** Definition of the groups based on magnetic resonance imaging (MRI) findings, following the classification used in our previous study [30].

Group	n	Definition (MRI Findings)
OC/DD (−) group	59	Articular discs in normal position bilaterally, with no degenerative osseous change in either mandibular condyle
UDD group	53	Unilateral disc displacement, with no degenerative osseous change in either mandibular condyle
BDD group	89	Bilateral disc displacement, with no degenerative osseous change in either mandibular condyle
OA group	39	Degenerative osseous change in one or both mandibular condyles (flattening, erosion, osteophyte formation, or sclerosis)

Degenerative osseous change was judged according to the Research Diagnostic Criteria for Temporomandibular Disorders (RDC/TMD) image analysis criteria [12]. OC/DD (−) = Osseous Change/Disc Displacement negative; UDD = unilateral disc displacement; BDD = bilateral disc displacement; OA = osteoarthritis.

**Table 2:** Age and distribution of TMJ pain by MRI-based structural classification (four groups).

Group	Age (Median [IQR])	TMJ Pain Present, n (%)	TMJ Pain Absent, n (%)	Total	χ^2^	df	*p*
OC/DD (−) group	22.5 [19.0–29.0]	17 (28.8)	42 (71.2)	59			
UDD group	22.0 [19.0–28.5]	22 (41.5)	31 (58.5)	53	2.359	3	0.501
BDD group	22.0 [18.8–30.0]	28 (31.5)	61 (68.5)	89			
OA group	25.5 [20.0–31.3]	14 (35.9)	25 (64.1)	39			
Total	22.0 [19.0–30.0]	81 (33.8)	159 (66.2)	240			

TMJ pain data are presented as n (%). χ^2^ = Pearson’s chi-squared statistic; df = degrees of freedom. IQR = interquartile range. Age is presented as median [interquartile range] and was compared among the four groups using the Kruskal–Wallis test (H = 1.686, df = 3, *p* = 0.640). TMJ pain was defined as familiar pain elicited on mandibular opening, lateral or protrusive movement, or palpation of the TMJ according to the DC/TMD Axis I examination.

**Table 3:** OBC total score and JFLS score by MRI-based structural classification.

Measure	OC/DD (−) Group (n = 59)	UDD Group (n = 53)	BDD Group (n = 89)	OA Group (n = 39)	H	*p*	ε^2^	JT p	OA vs. OC/DD (−)
OBC total score (0–84)	18 [14–22]	19 [14–24]	19 [14–23]	22 [17.5–29.5]	8.719	0.033	0.024	0.011	*
JFLS score (0–10)	0.10 [0–0.78]	0.40 [0–1.20]	0.25 [0–1.05]	0.70 [0.08–1.85]	7.710	0.052	0.020	0.016	—
Subjects with JFLS score of 0 (%)	45.8	35.8	30.3	23.1	—	—	—	—	—

Values are median [interquartile range]. The JFLS score was calculated as the mean of the 20 items in accordance with the standard scoring procedure for the JFLS-20 [15,16]. JT = Jonckheere–Terpstra test based on the prespecified order OC/DD (−) → UDD → BDD → OA. ε^2^ = epsilon squared. * adjusted *p* < 0.05.

**Table 4:** Proportion of subjects with a high oral behavior score (OBC total score ≥ 25) and odds ratios with the OC/DD (−) group as the reference.

Group	OBC ≥ 25, n (%)	OR	95% CI	*p*
OC/DD (−) group (n = 59)	12 (20.3)	1 (reference)	—	—
UDD group (n = 53)	9 (17.0)	0.80	0.31–2.09	0.650
BDD group (n = 89)	20 (22.5)	1.14	0.51–2.54	0.758
OA group (n = 39)	16 (41.0)	2.72	1.11–6.70	0.029
Trend (group as an ordinal score 1–4)	—	1.37	1.02–1.85	0.037

Secondary analysis by binary logistic regression, based on the same data as the main analysis (Table 3). OR = odds ratio; CI = confidence interval. The cut-off of 25 follows the DC/TMD scoring manual and the OPPERA studies [23,38].

**Table 5:** Item-level intergroup comparison of the OBC, the JFLS, and oral function.

Variable	OC/DD (−) Group (n = 59)	UDD Group (n = 53)	BDD Group (n = 89)	OA Group (n = 39)	H	*p*	ε^2^	OA vs. OC/DD (−)	OA vs. UDD	OA vs. BDD
OBC item 4	0.61 ± 0.89	0.60 ± 0.88	0.80 ± 1.01	1.21 ± 1.06	11.211	0.011	0.035	*	*	n.s.
JFLS item 2	1.58 ± 2.49	1.96 ± 2.72	1.93 ± 2.96	3.36 ± 3.54	7.961	0.047	0.021	*	n.s.	n.s.
JFLS item 5	0.27 ± 1.10	0.36 ± 1.29	0.39 ± 1.51	0.79 ± 1.91	8.571	0.036	0.024	*	n.s.	n.s.
Occlusal contact area (mm^2^)	17.08 ± 10.57	14.30 ± 7.30	13.85 ± 6.39	11.59 ± 5.90	8.064	0.045	0.021	*	n.s.	n.s.
Maximum occlusal force (N)	631.36 ± 337.33	562.67 ± 245.23	550.30 ± 252.19	468.95 ± 185.98	5.623	0.131	0.011	n.s.	n.s.	n.s.
Occlusal force per unit area (N/mm^2^)	39.59 ± 8.72	41.23 ± 7.72	40.64 ± 7.39	42.80 ± 9.60	3.895	0.273	0.004	n.s.	n.s.	n.s.

Values are mean ± standard deviation. Kruskal–Wallis test (*df* = 3); pairwise comparison by Dunn’s post hoc test with Bonferroni correction. In addition to the significant variables, maximum occlusal force and occlusal force per unit area are shown because they are addressed in the Discussion. * *p* < 0.05; n.s. = not significant. ε^2^ = epsilon squared. OBC item 4 = clenching teeth while awake; JFLS item 2 = chewing hard bread; JFLS item 5 = chewing soft food. Occlusal contact area and maximum occlusal force were strongly correlated (Pearson r = 0.934).

**Table 6:** Correlations between the Axis II measures and the oral function parameters.

Variable	Occlusal Contact Area r (*p*)	Maximum Occlusal Force r (*p*)	Tongue Pressure r (*p*)	Lip-Closing Force r (*p*)	Masticatory Performance ΔE r (*p*)
OBC item 1	−0.06 (0.37)	−0.05 (0.46)	0.05 (0.41)	0.04 (0.58)	0.18 (<0.01) **
OBC item 4	−0.03 (0.63)	0.01 (0.87)	0.05 (0.46)	0.12 (0.07)	0.15 (0.02) *
OBC item 5	−0.11 (0.10)	−0.05 (0.47)	0.02 (0.72)	−0.03 (0.61)	0.13 (0.05) *
OBC item 13	0.01 (0.82)	0.04 (0.56)	0.09 (0.18)	0.16 (0.01) *	0.09 (0.16)
OBC item 15	0.14 (0.02) *	0.17 (<0.01) **	0.01 (0.83)	−0.14 (0.03) *	0.05 (0.48)
OBC item 19	0.06 (0.38)	0.05 (0.46)	0.03 (0.60)	−0.13 (0.04) *	−0.07 (0.28)
JFLS item 1	−0.17 (<0.01) **	−0.16 (0.01) *	−0.04 (0.50)	0.10 (0.11)	0.02 (0.76)
JFLS item 2	−0.14 (0.03) *	−0.14 (0.03) *	−0.04 (0.51)	0.11 (0.08)	0.03 (0.67)
JFLS item 3	−0.24 (<0.01) **	−0.21 (<0.01) **	−0.02 (0.79)	0.11 (0.09)	0.02 (0.80)
JFLS item 4	−0.11 (0.09)	−0.14 (0.04) *	−0.09 (0.16)	0.07 (0.31)	0.03 (0.61)
JFLS item 5	−0.16 (0.01) *	−0.16 (0.01) *	0.03 (0.66)	0.10 (0.11)	0.04 (0.52)
JFLS item 6	−0.01 (0.92)	−0.03 (0.63)	−0.03 (0.65)	0.26 (<0.01) **	−0.05 (0.45)

r = Pearson’s product-moment correlation coefficient. * *p* < 0.05; ** *p* < 0.01. ΔE = color change during free chewing. Items shown are those with a significant unadjusted correlation (*p* < 0.05) with at least one oral function parameter; all items are listed in Supplementary Table S2. Asterisks are based on unrounded *p* values (OBC item 5 × masticatory performance, *p* = 0.047).

## Data Availability

All of clinical data are available in the Supplementary Materials.

## Supplementary Materials

The following supporting information can be downloaded at: https://www.mdpi.com/article/10.3390/dj14100660/s1, File S1: All the data analyzed in this study; Table S1: Item-level intergroup comparison of all OBC and JFLS items and oral function parameters; Table S2: Correlations between all OBC and JFLS items and the oral function parameters.

## Abbreviations

The following abbreviations are used in this manuscript:

TMD	Temporomandibular disorder
TMJ	Temporomandibular joint
DC/TMD	Diagnostic Criteria for Temporomandibular Disorders
MRI	Magnetic resonance imaging
OBC	Oral Behaviors Checklist
JFLS	Jaw Functional Limitation Scale
OA	Osteoarthritis
BDD	Bilateral disc displacement
UDD	Unilateral disc displacement
OC/DD (−)	Osseous Change/Disc Displacement negative
OR	Odds ratio
CI	Confidence interval
IQR	Interquartile range
